# Increased HLA-DR Expression on M2a Monocytes and Helper T Cells in Patients with COPD and Asthma–COPD Overlap Contributes to Disease Severity via Apoptosis and ROS

**DOI:** 10.3390/antiox14121507

**Published:** 2025-12-16

**Authors:** Yung-Che Chen, Kuo-Tung Huang, Chiu-Ping Lee, Po-Yuan Hsu, Yu-Ping Chang, Chao-Chien Wu, Sum-Yee Leung, Chang-Chun Hsiao, Meng-Chih Lin

**Affiliations:** 1 Division of Pulmonary and Critical Care Medicine, Department of Medicine, Kaohsiung Chang Gung Memorial Hospital, Chang Gung University College of Medicine, Kaohsiung 83301, Taiwan; yungchechen@yahoo.com.tw (Y.-C.C.); jelly@adm.cgmh.org.tw (K.-T.H.); choupeen@gmail.com (C.-P.L.); wanpasum@cgmh.org.tw (P.-Y.H.); b9002087@cgmh.org.tw (Y.-P.C.); my47104710@gmail.com (C.-C.W.); sumyeeleung@hotmail.com (S.-Y.L.); 2 Graduate Institute of Clinical Medical Sciences, Department of Medicine, College of Medicine, Chang Gung University, Taoyuan 33302, Taiwan; 3 Department of Respiratory Therapy, Kaohsiung Chang Gung Memorial Hospital, Chang Gung University College of Medicine, Kaohsiung 83301, Taiwan; 4 Division of Pulmonary and Critical Care Medicine, Kaohsiung Municipal Ta-Tung Hospital, Kaohsiung 801735, Taiwan

**Keywords:** asthma, chronic obstructive pulmonary disease, HLA-DR, HLA-DQ

## Abstract

Objective: Ongoing debates focus on the role of human leukocyte antigen (HLA) class II expression in shaping clinical phenotypes of chronic inflammatory airway diseases. This study seeks to clarify the impact of class II HLA on chronic obstructive pulmonary disease (COPD) and asthma–COPD overlap (ACO). Method: The expression levels of HLA-DQ/DR in blood immune cells were analyzed in 116 participants: 41 with COPD, 37 with ACO, 20 with pure asthma, and 18 healthy subjects (HS). Results: In the COPD group, HLA-DR protein expression levels were significantly elevated on blood M2a monocytes (7695 ± 3743 vs. 5391 ± 3153 MFI, *p* = 0.026), helper T cells (2551 ± 956 vs. 1836 ± 531 MFI, adjusted *p* = 0.018), cytotoxic T cells (1591 ± 531 vs. 1360 ± 477 MFI, adjusted *p* = 0.036), and B cells (20,667 ± 7985 vs. 15,694 ± 2003 MFI, adjusted *p* = 0.031) compared to the HS group. Conversely, no significant changes were observed in the asthma group. In ACO patients, helper T cells showed increased HLA-DR protein expression (2416 ± 914 MFI; adjusted *p* = 0.016) compared with the HS group. Higher levels of HLA-DR expression correlated with reduced pulmonary function, frequent exacerbations, and more severe symptoms. Following one year of treatment in 14 COPD and 16 ACO patients, HLA-DR protein expression on blood helper T cells, cytotoxic T cells, M2a monocytes, and neutrophils significantly declined (all *p* < 0.05). In vitro experiments demonstrated that exposure of M2- or M1-polarized THP-1 cells to a stimulus mix containing cigarette smoke extract, house dust mite antigens, and lipopolysaccharide led to up-regulation of HLA-DR expression. This response was linked to increased apoptosis and reduced production of reactive oxygen species. Conclusions: Up-regulation of HLA-DR in COPD and ACO patients may represent a novel biomarker for assessing disease severity and treatment response. Additionally, it could serve as a useful tool to distinguish COPD and ACO from asthma.

## 1. Introduction

Asthma and chronic obstructive pulmonary disease (COPD) are the two most prevalent obstructive airway conditions. Asthma–COPD overlap (ACO) is identified by persistent airflow limitation along with characteristics of both conditions. Numerous studies have highlighted that ACO patients experience a heavier symptom burden and more frequent and severe exacerbations compared to those with asthma or COPD individually. These patients often endure a lower quality of life and face higher healthcare expenses in comparison to those diagnosed with either condition alone [1,2,3]. As of 2016, asthma and COPD were estimated to impact approximately 339 million and 251 million individuals worldwide, respectively, while ACO may affect around 29.6% of COPD cases and 26.5% of asthma cases globally [4]. Unfortunately, patients with ACO are frequently excluded from asthma and COPD clinical trials, and the scarcity of specific interventional research on ACO has led to a significant gap in understanding effective diagnostic biomarkers and treatment strategies for this condition.

Asthma typically involves airway hyperresponsiveness, causing reversible airflow obstruction driven by type 2 inflammation characterized by eosinophils. On the other hand, COPD manifests as a progressive and irreversible obstruction of airflow, usually triggered by smoking or biomass exposure, and it is associated with systemic inflammation involving neutrophils, CD8^+^ T cells, and macrophages. These elements contribute to airway remodeling, airflow limitation, symptoms, and microorganism colonization [5,6,7]. While these distinct inflammatory mechanisms overlap in ACO patients, no definitive biomarker or biomarker combination currently exists to accurately identify ACO. Research has indicated that ACO patients exhibit elevated exhaled nitric oxide levels, higher blood eosinophil counts, and type 2 helper T cell markers, as well as increased immunoglobulin E (IgE) levels compared with COPD-only patients [1,8]. Nevertheless, the specific gene expression profiles distinguishing ACO from asthma or COPD remain unexplored. Additionally, little is known about the innate immune responses or their influence on airway remodeling in ACO.

The human leukocyte antigen (HLA), also termed the major histocompatibility complex (MHC), plays a crucial role in immune regulation. Encoded by the HLA gene complex located on the short arm of chromosome 6 within a dense genetic region of about 7.6 Mb, it includes over 250 genes and exhibits the highest degree of polymorphism among human chromosomal regions [9]. HLA class I molecules present foreign antigens to CD8^+^ cytotoxic T cells and are expressed on all nucleated cells. Meanwhile, HLA class II molecules—comprising HLA-DP, HLA-DQ, and HLA-DR—are responsible for presenting antigenic peptides to CD4^+^ helper T cells and are primarily expressed on antigen-presenting cells like T cells, B cells, neutrophils, monocytes, and eosinophils. Pro-inflammatory cytokines can stimulate MHC class II expression on these immune cells [10].

Long-term low-dose azithromycin therapy has been observed to reduce pro-inflammatory cytokine production, enhance macrophage phagocytosis, and increase anti-inflammatory cytokine expression in COPD. Interestingly, this effect also correlates with reduced expression of certain HLA class II molecules in the respiratory tract, such as HLA-DPA1, HLA-DRA, and HLA-DRB4 [11]. Specific *HLA* alleles have shown associations with COPD, particularly susceptibility to early-stage COPD [12,13,14]. Moreover, increased HLA-DQA1 and HLA-DR expressions were both associated with COPD frequent exacerbators, while serum soluble HLA-II molecule has been shown to be negatively correlated with values of forced expiratory volume in 1 s (FEV1) and FEV1/forced vital capacity (FVC) ratio in COPD patients. Higher HLA-DQA1 and HLA-DR expression levels have been associated with frequent exacerbators in COPD patients, while serum-soluble HLA class II molecules have shown negative correlations with lung function indices such as forced expiratory volume in 1 s (FEV1) value and FEV1/forced vital capacity (FVC) ratios. In contrast, decreased expressions of HLA-DQA1 and HLA-DQB1 genes in blood leukocytes have been tied to COPD exacerbations [15,16,17,18]. In asthma, research has also highlighted strong links between specific *HLA* alleles and disease susceptibility or immune regulation pathways [19,20,21,22,23,24].

Recent research utilizing an animal model has shown that ACO mice exhibit significantly more extensive inflammation compared to those with COPD or asthma, with antigen presentation pathways, such as HLA-DRA, playing a key role [25]. The absence of a clearly defined ACO classification underscores the importance of identifying specific biomarkers that can aid in distinguishing this phenotype. Targeting this subgroup of patients is crucial for advancing mechanistic studies and facilitating tailored therapeutic approaches. Current investigations into HLA-DR/DQ expression across various immune cells in COPD or asthma patients remain scarce, and debates continue regarding the influence of HLA class II expression on the clinical characteristics of COPD, asthma, and ACO. Furthermore, it has been established that down-regulated HLA-DR correlates with diminished monocyte responsiveness and poor outcomes in sepsis. In stable COPD, where pulmonary microorganism colonization is prevalent in approximately 40% of cases, these conditions are linked to frequent exacerbations and progressive lung function deterioration [26,27].

This study aimed to gain a deep understanding of the role of HLA Class II molecules in asthma, COPD, and ACO, and to determine whether HLA-DQ/DR expression can be used to discriminate among these three chronic inflammatory airway diseases or to predict their clinical outcomes. Thus, we measured HLA-DQ/DR protein and gene expression in blood immune cells by flow cytometry and quantitative reverse transcriptase polymerase chain reaction (RT-PCR), respectively, in a cohort of 18 healthy subjects (HS), 41 COPD alone patients, 37 ACO patients, and 20 asthma alone patients. Meanwhile, in vitro cell culture models using THP-1 cells and blood leukocytes from healthy subjects were used to verify the biological functions of HLA-DQ/DR molecules in response to cigarette smoke extract (CSE), house dust mite (HDM), lipopolysaccharide (LPS), and their mixtures. *HLA-DQ/DR* genetic polymorphism has been shown to be linked to the susceptibility of asthma or COPD. However, ongoing debates focus on the role of HLA class II expression in shaping clinical phenotypes of chronic inflammatory airway diseases. The study aimed to determine whether HLA-DR/DQ expression varied among patients with COPD, asthma, and ACO, and to clarify the relationship between HLA-DR/DQ expression and clinical outcomes, including pulmonary function, endurance, symptom burden, and exacerbations.

## 2. Materials and Methods

### 2.1. Study Subjects

**Figure 1:**
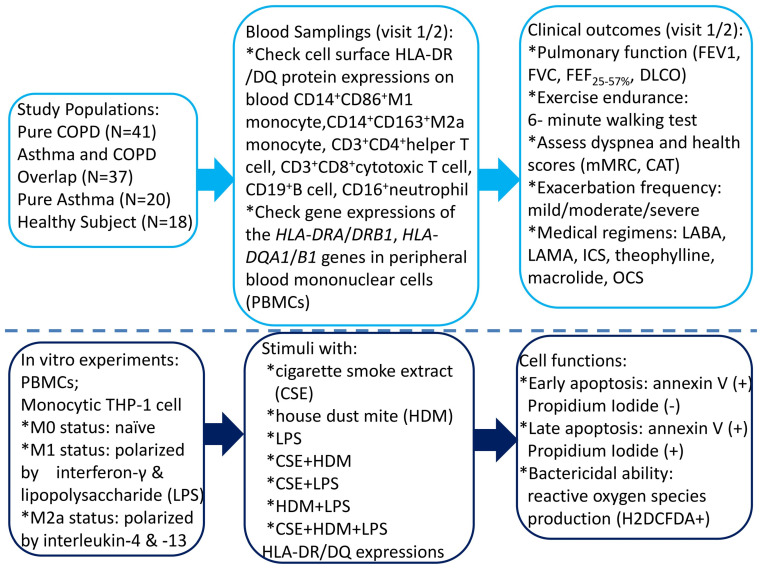
A flowchart illustrating the graphical representation of all the tasks carried out, along with their logical sequence. COPD = chronic obstructive pulmonary disease; HLA = human leukocyte antigen; CAT = COPD Assessment Test; mMRC = modified Medical Research Council; LABA = long-acting β2 agonist; LAMA = long-acting muscarinic antagonist; ICS = inhaled corticosteroid; OCS = oral corticosteroid; DLCO = diffusion capacity of carbon monoxide; FEV1 = forced expiratory volume in 1 s; FVC = forced vital capacity; FEF_25–75%_ = forced mid-expiratory flow.

This study was conducted in accordance with the Declaration of Helsinki and approved by the Institutional Review Board of Chang Gung Memorial Hospital, Taiwan (certificate number: 202102182B0; Approval Date: 6 January 2022). The study participants were recruited from the pulmonary clinics of Kaohsiung Chang Gung Memorial Hospital from January 2023 through July 2024. All the participants were of Taiwanese Han ancestry. Written informed consent was obtained from each subject participating in the study. Figure 1 shows the graphical representation of all the tasks we performed and their logical sequence.

The inclusion criteria for COPD and the definition of acute exacerbation were in accordance with the Global Initiative for Chronic Obstructive Lung Disease (GOLD) 2022 guidelines [3]. Briefly, an exacerbation of COPD was defined as an event characterized by increased dyspnea, cough, or sputum that worsens in <14 days, while its severity was defined by the treatment that is required (mild: short-acting bronchodilator (BD) only; moderate: antibiotics or systemic steroid; severe: hospitalization or a visit to the emergency department). The inclusion criteria for COPD patients included an age of more than 40 years, a history of 10 or more pack-years of smoking, a ratio of FEV1 to FVC of 70% or less after BD use by standard lung function test, and a stable clinical condition with no acute exacerbations for at least 4 weeks prior to enrollment. The COPD Assessment Test (CAT) and the Modified Medical Research Council (mMRC) scales were used to assess health status and the degree of dyspnea, respectively [28,29].

The inclusion criteria for asthma patients were based on the definition of American Thoracic Society, including fluctuating symptoms of wheezing, cough, chest tightness, or dyspnea relieved by an inhalation β2-agonist drug and corticosteroids according to Global Initiative for Asthma (GINA) guidelines and classified according to their severity of symptoms and spirometry (pre-BD FEV1/FVC, FEV1% predicted, BD response) [30].

The major criteria for ACO included: (1) Persistent airflow limitation [post-BD FEV1/FVC < 0.7] in individuals 40 years of age or older; (2) At least 10 pack-years of tobacco smoking; (3) Documented history of asthma before 40 years of age or BD response of FEV1 ≥ 200 mL and 12% from baseline values on two or more visits; (4) Peripheral blood eosinophil count of ≥300 cells/μL or serum IgE level ≥ 150 IU/mL [31].

The inclusion criteria for HS included: (1) Age ≥ 40 years; (2) former smoker or non-smoker; (3) Normal pulmonary function test: FEV1/FVC ≥ 0.7, FEV1 ≥ 80% predicted, FVC ≥ 80% predicted, 6-min walking test (6MWT) distance ≥ 80% predicted, alveolar volume (VA) ≥ 80% predicted, and diffusion capacity of carbon monoxide (DLCO) ≥ 80% predicted. Exclusion criteria included (apply to COPD, asthma, ACO, and HS): (1) Age < 40 years; (2) Recent exacerbation: hospitalization, antibiotic use, or increased corticosteroid use (oral or inhaled) for increased symptoms of COPD within 30 days; (3) Pulmonary hospitalization for diseases other than exacerbation within the past 4 weeks (such as pneumonia); (4) Other concomitant chronic respiratory disorders; (5) Autoimmune diseases.

### 2.2. Blood Sample Collection

Fresh venous blood samples of 20 mL were collected from individuals with COPD, asthma, ACO, and HS at the first enrollment (visit one). For COPD and ACO patients who underwent 1 year of treatment according to GOLD and GINA guidelines, another blood sample was obtained at a follow-up visit (visit 2). Peripheral blood mononuclear cells (PBMCs) were isolated through Ficoll–Histopaque density gradient centrifugation using Histopaque solutions (1.077 and 1.119; Sigma Diagnostics, St. Louis, MO, USA). The cells were stored at −80 °C until RNA isolation.

### 2.3. Determining Cell Surface Protein Expressions of MHC Class II of Peripheral Blood CD14^+^ Monocyte, CD16^+^Neutrophil, CD125^+^Siglec8^+^ Eosinophil, CD19^+^ B Cell, CD3^+^CD4^+^ Helper T (Th) Cell, and CD3^+^CD8^+^Cytotoxic T (Tc) Cell by Flowcytometry

Surface protein expression for MHC Class II molecules on different immune cells was assessed using flow cytometry. Monoclonal antibodies were directly conjugated for simultaneous staining over 30 min. The analyzed immune cell markers included CD3^+^ (PE-BV510 Mouse Anti-Human), CD4^+^ (APC Mouse Anti-Human), CD8^+^ (APC-Cy™7 Mouse Anti-Human), CD16^+^ (PC7), CD14^+^ (APC-Cy™7 Mouse Anti-Human), CD163^+^ (PE Mouse Anti-Human), CD86^+^ (APC Mouse Anti-Human), CD19^+^ (PE Mouse Anti-Human), CD125^+^Siglec8^+^ eosinophils, and specific helper T (Th) and cytotoxic T (Tc) cells. MHC class II markers encompassed BV421 Mouse Anti-Human HLA-DQ, APC Mouse Anti-Human HLA-DR, BB515 Mouse Anti-Human HLA-DR, BV421 Mouse IgG2a, BV510 Mouse IgG1 (BD Horizon™), PE Mouse IgG1, and APC Mouse IgG1 (BD Pharmingen™). Analysis of 20,000 events was performed on whole blood by using BD LSR II Flow Cytometer (Becton, Dickinson and Company, Franklin Lakes, NJ, USA) and BD FACSDiva^TM^ 8.0.2 software. HLA-DQ/DR protein expression was defined as the percentage of stain-positive cells or mean fluorescence intensity (MFI), which was corrected for background fluorescence with the corresponding isotype controls.

### 2.4. Determination of HLA-DQ/DR Gene Expressions of PBMC Samples by Quantitative Real-Time Reverse Transcriptase–Polymerase Chain Reaction (RT-PCR)

RNA was extracted from PBMCs or THP-1 cells using Trizol^®^ Reagent (Invitrogen, Carlsbad, CA, USA). A standardized quantity of total RNA (500 ng) from each sample, performed in triplicate, was reverse-transcribed into cDNA using the High-Capacity cDNA Reverse Transcription Kit (Thermo Fisher Scientific, Waltham, MA, USA) with random hexamer primers in a 20 μL reaction setup as per the manufacturer’s protocol. Primer sequences for qRT-PCR assays targeting *HLA-DRA*, *HLA-DRB1*, *HLA-DQA1*, and *HLA-DQB1* genes are included in Supplementary Table S1. GAPDH was employed as a housekeeping gene for normalization of expression levels. PCR assays were conducted using the Applied Biosystems 7500 Fast Real-Time PCR System (Thermo Fisher Scientific), with reactions containing Fast SYBR Master Mix, diluted cDNA, and appropriate gene-specific primers in a 10 μL final volume. Thermal cycling conditions included an initial denaturation step at 95 °C for 20 s, followed by 40 cycles of 95 °C for 3 s and 60 °C for 30 s. The ∆∆Ct method was used to calculate relative gene expression, with median control group values serving as calibrators.

### 2.5. In Vitro Human Monocytic THP-1 and PBMC Culture Models Under CSE, HDM, and LPS Stimulation

#### 2.5.1. M1/M2 Polarization Protocol

PBMCs from 4 HS and the human monocytic THP-1 cell line were exposed to CSE (2.5%/mL), HDM extract (Dermatophagoides pteronyssinus 25 mg/mL plus Dermatophagoides farinae 25 mg/mL), LPS (50 ng/mL), or a mixture. Control cells were maintained in normal culture medium conditions. Protein expressions of HLA-DQ/DR and gene expression levels of the HLA-DRA/DRB1/DQA1/DQB1 genes were determined using flow cytometry and quantitative RT-PCR methods, respectively.

THP-1 cells were obtained from ATCC (Manassas, VA, USA) and cultured in RPMI 1640 medium (Thermo Fisher Scientific) with 5.5 mM glucose and containing 10% fetal bovine serum, and supplemented with 1% antibiotics (100 U/mL penicillin, 100 mg/mL streptomycin). THP-1 cells were either left untreated (M0), polarized towards M1 cells by adding 50 ng/mL interferon-γ and 50 ng/mL LPS for 24 h, or towards M2 cells via the addition of 25 ng/mL IL-4 and 25 ng/mL IL-13 for 24 h.

#### 2.5.2. Measurement of Cell Apoptosis by Flow Cytometry Analysis

Apoptosis proportions were assessed through flow cytometry using the Annexin V/Propidium Iodide (PI) apoptosis detection kit from BD Biosciences (Franklin Lakes, NJ, USA). Post-treatment, cells were incubated with 5 µL FITC-Annexin V and 5 µL PI for 15 min at room temperature. Cell staining results were analyzed using the FACScan flow cytometry system (Becton Dickinson, San Diego, CA, USA).

#### 2.5.3. Measurement of Intracellular Reactive Oxygen Species (ROS)

Cells seeded at a density of 1 × 10^6^ cells/mL were treated with 2′,7′-dichlorodihydrofluorescein diacetate (H2DCFDA; catalog no. D6883; Sigma Aldrich, St. Louis, MO, USA). The fluorescence intensity associated with ROS levels was measured via flow cytometry on the FL1 channel with excitation and emission wavelengths set to 488 nm and 535 nm, respectively. Measurements were carried out using the Cytomics™ FC500 (Beckman Coulter, Brea, CA, USA).

### 2.6. Statistical Analysis

Continuous values were presented as the mean ± standard deviation (SD). Kolmogorov–Smirnov test and the Anderson–Darling test were used to assess whether the dataset follows a normal distribution. One-way analysis of variance ANOVA test followed by post hoc analysis with the Bonferroni test was used for comparing mean values of more than two clinical groups in case of normal and homogeneous data. In contrast, the Brown–Forsythe test, followed by post hoc analysis with Tamhane’s T2 test, was used for normal and non-homogeneous data. The Kruskal–Wallis H test followed by post hoc analysis with Dunn’s test was used to compare median values across more than two in vitro experimental groups and the clinical groups, in cases of non-normal data or detected outliers. The Wilcoxon signed-rank test was used to compare median values between the COPD and ACO groups before and after 1 year of medical treatment. Categorical variables were analyzed using the Chi-square test. Continuous variables that showed significant differences in subgroup comparisons were included in a multivariate linear regression analysis to adjust for potential confounding factors, including age, sex, body mass index, and Charlson comorbidity index, and to obtain adjusted *p*-values. Pearson and Spearman’s correlation tests were used to analyze the correlation coefficient (R) between two continuous variables in the clinical and in vitro experimental groups, respectively. All tests are two-tailed, and the null hypothesis is rejected at *p* < 0.05. All analyses were performed using SPSS software version 22.0 (SPSS Corp., Chicago, IL, USA).

## 3. Results

### 3.1. Demographic and Baseline Characteristics in the Study Cohort

Table 1 and Supplementary Table S2 outline the demographic and baseline characteristics of the four groups studied: 41 patients with COPD only, 37 patients with ACO, 20 patients with asthma only, and 18 healthy subjects (HS). Compared to asthma patients and HS, individuals with COPD and ACO were older, predominantly male, and had greater exposure to cigarette smoke (all *p* < 0.05). ACO patients also demonstrated a higher Charlson Comorbidity Index compared to the other three groups.

Pulmonary function metrics revealed that COPD and ACO patients had lower FEV1% predicted values, FEV1/FVC ratios, and FEF_25–75%_ % predicted values (maximum mid-expiratory flow) compared to asthma patients and HS (all *p* < 0.05). However, these parameters were comparable between the COPD and ACO groups. COPD patients exhibited reduced DLCO values and alveolar volume in comparison to HS. The ACO group experienced more total exacerbation events, including moderate exacerbations, within the past year compared to the COPD-only or HS groups (*p* < 0.05; see Supplementary Figure S1A).

Symptom scores indicated that ACO patients had higher CAT scores than those in the COPD or HS groups (Supplementary Figure S1B). Additionally, they presented an elevated mMRC symptom score relative to HS. Both ACO and asthma patients had higher absolute eosinophil counts compared to COPD-only patients and HS (all *p* < 0.05; Supplementary Figure S1C).

### 3.2. Increased HLA-DR Protein Expressions of Blood Helper T Cell and M2a Monocyte in COPD and ACO Patients

**Figure 2:**
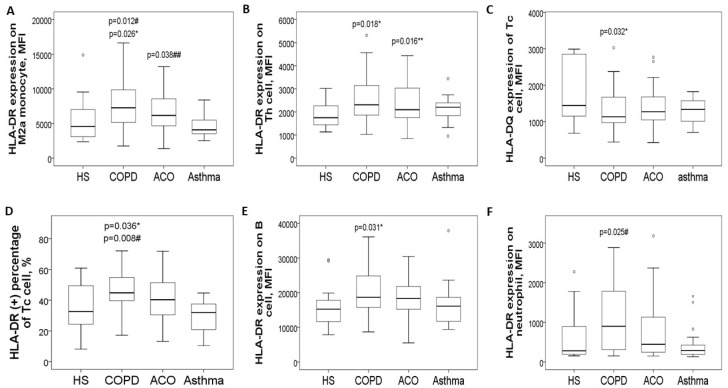
HLA-DR/DQ protein expression in blood immune cells at diagnosis, stratified into four groups. (**A**) HLA-DR protein expression on blood M2a monocytes was increased in pure COPD patients versus either HS or asthma, and also up-regulated in ACO patients versus asthma. (**B**) HLA-DR protein expression on blood helper T cells was increased in both pure COPD and ACO patients versus HS. (**C**) HLA-DQ protein expression on cytotoxic T cells was decreased in both pure COPD and ACO patients versus HS. (**D**) HLA-DR protein expression on cytotoxic T cells was increased in pure COPD patients versus either the HS or asthma group. (**E**) HLA-DR protein expression on B cells was increased in pure COPD patients versus HS. (**F**) HLA-DR protein expression on neutrophils was increased in pure COPD patients versus asthma patients. * compared between chronic obstructive pulmonary disease (COPD) and healthy subjects (HS). ** compared between asthma–COPD overlap (ACO) and HS. ^#^ compared between COPD and asthma. ^##^ compared between ACO and asthma.

**Figure 3:**
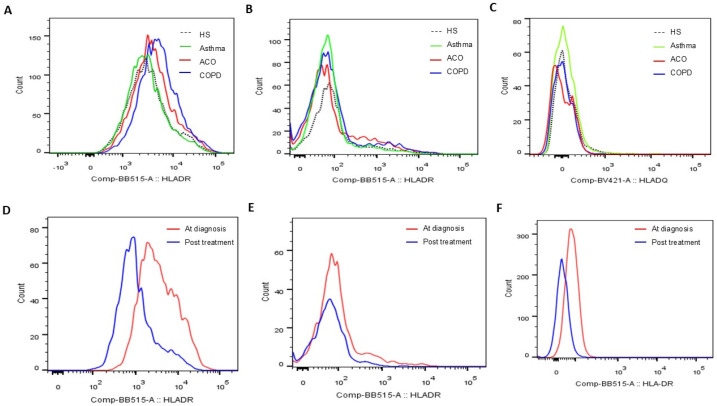
Representative histograms showing (**A**) HLA-DR protein expression determined on blood CD14^+^CD163^+^ M2a populations, (**B**) HLA-DR protein expression on blood CD3^+^CD4^+^ helper T cell populations, and (**C**) HLA-DQ protein expression on blood CD3^+^CD8^+^ cytotoxic T cell populations at the first study enrollment (visit 1). Representative histograms showing HLA-DR protein expression determined on blood (**D**) CD14^+^CD163^+^ M2a, (**E**) CD3^+^CD4^+^ helper T cell, and (**F**) CD16^+^ neutrophil populations before and after 1-year medical treatment (visits 1 and 2).

The expression levels of HLA-DR proteins on blood CD14^+^CD163^+^ M2a monocytes (Figure 2A and Figure 3A) were significantly elevated in the COPD-only group (7695 ± 3743 MFI) compared to the HS group (5391 ± 3153 MFI, adjusted *p* = 0.026) and the asthma group (4716 ± 1679 MFI, adjusted *p* = 0.012). Additionally, these levels were higher in the ACO group (6782 ± 2718 MFI; adjusted *p* = 0.038) than in the asthma group. Similarly, HLA-DR protein expression in CD3^+^CD4^+^ Th cells (Figure 2B and Figure 3B) was increased in both the COPD-only group (2551 ± 956 MFI, adjusted *p* = 0.018) and the ACO group (2416 ± 914 MFI, adjusted *p* = 0.016) compared to the HS group (1836 ± 531 MFI).

Conversely, HLA-DQ protein levels on blood CD3^+^CD8^+^ Tc cells were lower in both the COPD-only group (1320 ± 585 MFI) and the ACO group (1468 ± 740 MFI) compared to the HS group (2892 ± 3216 MFI, Figure 2C and Figure 3C). For HLA-DR expression in CD3^+^CD8^+^ Tc cells (Figure 2D), levels were significantly elevated in the COPD-only group (1591 ± 531 MFI) as opposed to the HS group (1360 ± 477 MFI, adjusted *p* = 0.036) or the asthma group (1438 ± 385 MFI, adjusted *p* = 0.008).

Moreover, HLA-DR protein expression in CD19^+^ B cells was higher in the COPD-only group (20,667 ± 7985 MFI, adjusted *p* = 0.031) compared with the HS group (15,694 ± 2003 MFI, Figure 2E). Similarly, elevated HLA-DR protein expression was noted in CD16^+^ neutrophils in the COPD-only group (1356 ± 1617 MFI, adjusted *p* = 0.025, Figure 2F) relative to the asthma group (445 ± 436 MFI). Supplementary Figure S2 provides representative two-color plots and histograms that identify various immune cell populations and display HLA-DR/DQ expression profiles in blood immune cells.

### 3.3. Negative Correlations Between HLA-DR Protein Expressions and Either Pulmonary Function Test Parameters or Exacerbation Frequency

**Figure 4:**
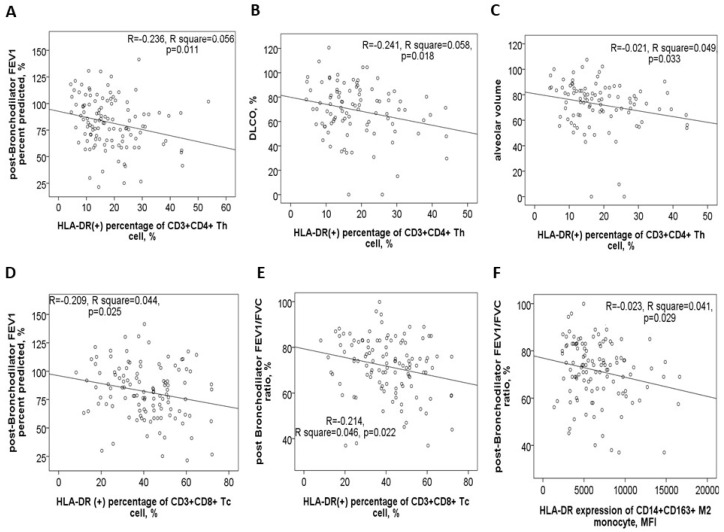
HLA-DR protein expression in blood immune cells correlates with clinical characteristics. HLA-DR expression of helper T cells was negatively correlated with (**A**) post-bronchodilator (BD) forced expiratory volume in 1 s (FEV1) %predicted value, (**B**) diffusion capacity of carbon monoxide (DLCO) %predicted value, and (**C**) alveolar volume %predicted value. HLA-DR expression of cytotoxic T cells was negatively correlated with (**D**) post-BD FEV1% predicted value and (**E**) post-BD FEV1/FVC ratio. (**F**) HLA-DR expression of M2a monocyte was negatively correlated with post-BD FEV1/FVC ratio.

In the 116 study participants, the HLA-DR protein expression on blood helper T cells showed a negative correlation with post-BD FEV1 values (Figure 4A), post-BD FEV1/FVC ratio, DLCO values (Figure 4B), and VA (Figure 4C). Conversely, it demonstrated a positive correlation with both CAT and mMRC symptom scores. Similarly, HLA-DR protein expression on blood cytotoxic T cells was negatively correlated with post-BD FEV1 values (Figure 4D), post-BD FEV1/FVC ratio (Figure 4E), and the percent predicted value of post-BD FEF_25–75%_. Meanwhile, this expression was positively linked to the mMRC symptom score. Additionally, HLA-DR expressions on both cytotoxic and helper T cells were found to positively correlate with the number of mild exacerbation events in the past year.

For blood M2a monocytes, HLA-DR protein expression exhibited a negative correlation with post-BD FEV1 values, post-BD FEV1/FVC ratio (Figure 4F), and the percent predicted value of post-BD FEF_25–75%_. Age also showed a positive correlation with HLA-DR expressions on Th, Tc, M2a, and M1 cells (Supplementary Figure S3A–D). Furthermore, cigarette smoke exposure, measured in pack-years, was positively associated with HLA-DR expression on Th, Tc, M2a, neutrophil, and B cells (Supplementary Figure S3E–I). To determine whether age, sex, smoking exposure, and comorbidities independently influenced HLA-DR protein expression, multivariable linear regression models were applied. The regression coefficients and confidence intervals are provided in Table 2.

### 3.4. Increased HLA-DRA and HLA-DRB1 Gene Expressions of PBMCs in COPD Patients

Gene expression levels of the *HLA-DRA* (1.01 ± 0.43 versus 0.67 ± 0.23-fold change, adjusted *p* = 0.019) and *HLA-DRB1* (1.42 ± 0.74 versus 0.81 ± 0.36-fold change, adjusted *p* = 0.011) genes were both elevated in the COPD-only group compared to the asthma-only group (see Supplementary Figure S4A,B). Additionally, *HLA-DRB1* gene expression showed a negative correlation with post-BD FEV1/FVC ratio, while *HLA-DQA1* gene expression was positively associated with DLCO % predicted values and negatively correlated with CAT symptom scores (see Supplementary Figure S4C–E). Gene expression levels of the *HLA-DRA* and *HLA-DRB1* genes also correlated positively with HLA-DR protein expressions on M1 and M2a (Supplementary Figure S4F), as well as with those on neutrophil and eosinophil. Similarly, *HLA-DQA1* and *HLA-DQB1* gene expressions were positively linked to HLA-DQ protein levels on Th, Tc, B, M1, and M2a cells (Supplementary Figure S4G), as well as on neutrophils.

### 3.5. Reduced HLA-DR Protein Expressions in COPD and ACO Patients After 1-Year Treatment

**Figure 5:**
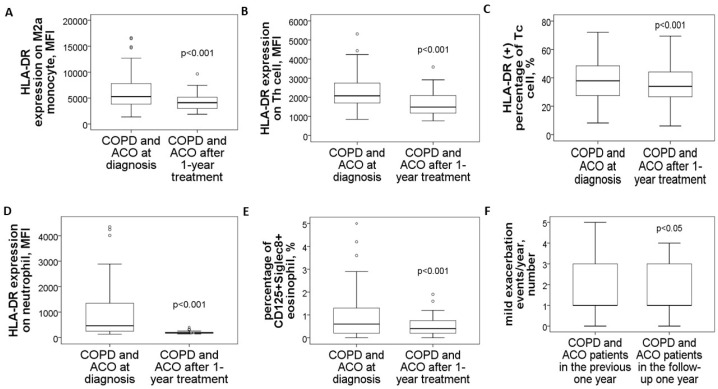
Changes in HLA-DR protein expressions of blood immune cells and clinical parameters in COPD and ACO patients after 1-year medical treatment. HLA-DR expressions of (**A**) blood M2a monocyte, (**B**) helper T cell, (**C**) cytotoxic T cell, and (**D**) neutrophil were reduced to the normal range after 1-year treatment according to the GOLD and GINA guidelines. (**E**) The percentage of CD125^+^Siglec8^+^ inflammatory eosinophils was reduced after 1-year treatment. (**F**) Mild exacerbation frequency was reduced during the follow-up year.

In a follow-up study involving 14 patients with pure COPD and 16 with ACO, blood samples collected after one year of medical treatment demonstrated significant reductions across various markers. M2a monocytes showed a marked decrease (4479 ± 1903 compared to 8242 ± 3593, *p* < 0.001, Figure 5A), as did HLA-DR expression on blood helper T cells (1649 ± 670 versus 2611 ± 956, *p* < 0.001, Figure 5B), cytotoxic T cells (% HLA-DR (+) Tc cells: 37 ± 14% versus 43 ± 12%, *p* < 0.001, Figure 5C), and neutrophils (198 ± 57 versus 1557 ± 1263, *p* < 0.001, Figure 5D). Similarly, the proportion of CD125^+^Siglec8^+^ inflammatory eosinophils significantly dropped (0.57 ± 0.48% compared to 1.92 ± 1.88%, *p* < 0.001, Figure 5E). Alongside these improvements, mild exacerbation events were reduced (1.4 ± 1.2 versus 2.1 ± 1.1 events, *p* = 0.012, Figure 5F).

### 3.6. Specific Medicines Prescribed for the 30 COPD or ACO Patients During the 1-Year Follow-Up Period

During the 1-year follow-up period, treatments administered to the 14 pure COPD and 16 ACO patients included a dual bronchodilator combining a long-acting muscarinic antagonist (LAMA) and a long-acting β2-agonist (LABA) in a single inhaler, and triple therapy consisting of LAMA, LABA, and inhaled corticosteroid (ICS) in one inhaler, or a combination of LABA and ICS in one inhaler plus LAMA in the other. Other medications included oral corticosteroids, theophylline, and macrolides such as azithromycin or erythromycin. Medicines prescribed for over six months were documented and analyzed (Table 3). It was observed that pure COPD patients received dual bronchodilators more frequently compared to ACO patients, while ACO patients were more commonly treated with triple therapy than pure COPD patients, both with statistically significant differences (*p* values < 0.05).

### 3.7. Negative Correlations Between HLA-DR Protein Expressions and Clinical Outcomes After 1-Year Medical Treatment

**Figure 6:**
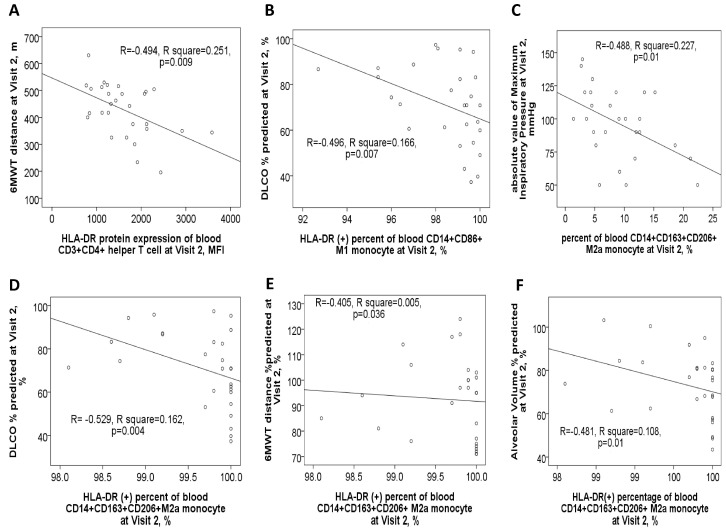
Correlations between clinical characteristics and HLA-DR protein expressions of blood immune cells in ACO and COPD patients after 1-year medical treatment (Visit 2). (**A**) HLA-DR expression of blood helper T cells was negatively correlated with the distance (meters) of the 6-min walking test. (**B**) HLA-DR expression of blood M1 monocytes was negatively correlated with DLCO %predicted values. HLA-DR expression of M2a monocyte was negatively correlated with (**C**) absolute value of maximum inspiratory pressure, (**D**) DLCO %predicted value, (**E**) 6-min walking distance %predicted value, and (**F**) alveolar volume %predicted value.

After one year of medical treatment (visit 2), patients with ACO continued to experience higher total and moderate exacerbation events compared to patients with pure COPD, as shown in Supplementary Figure S1D. At visit 2, the expression level of HLA-DR protein on Th cells was negatively linked to the 6MWT distance value (Figure 6A). Similarly, HLA-DR protein expression on M1 monocytes showed a negative correlation with DLCO% % predicted (Figure 6B). Furthermore, HLA-DR protein expression on M2a monocytes was negatively correlated with multiple pulmonary function parameters at visit 2, including the absolute value of maximum inspiratory pressure, DLCO% % predicted, 6MWT distance% % predicted, and alveolar volume% % predicted (Figure 6C–F).

### 3.8. In Vitro Experiments for Stimuli with CSE, HDM, LPS, or Their Mixture

#### 3.8.1. Increased HLA-DR/DQ Protein Expressions of Th and Tc Cells in Response to CSE Plus HDM Stimuli

**Figure 7:**
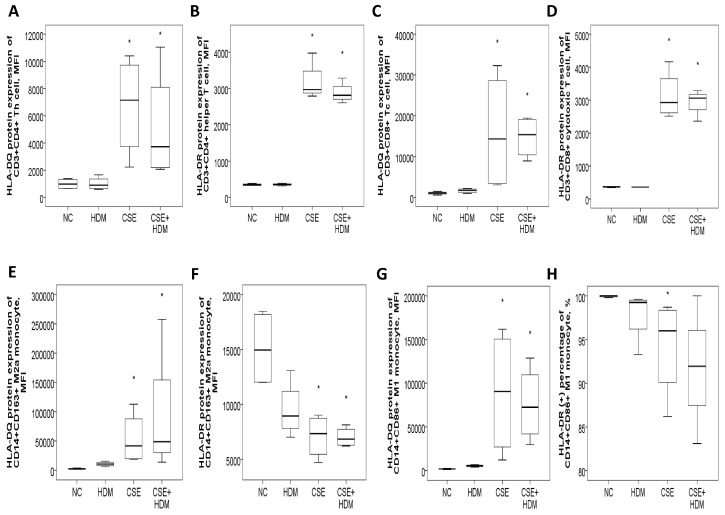
HLA-DR and HLA-DQ protein expressions of peripheral blood mononuclear cells from four healthy subjects in response to cigarette smoke extract (CSE) and house dust mite (HDM) stimuli in vitro. (**A**) HLA-DQ and (**B**) HLA-DR protein expressions of CD3^+^CD4^+^ helper T (Th) cells were both increased with CSE or CSE+HDM stimuli but not with HDM alone stimuli. (**C**) HLA-DQ and (**D**) HLA-DR protein expressions of CD3^+^CD8^+^ cytotoxic T (Tc) cells were both increased with either CSE or CSE+HDM stimuli, but not with HDM alone stimulus. (**E**) HLA-DQ protein expression of CD14^+^CD163^+^ M2a monocyte was increased with CSE or CSE+HDM stimuli, while (**F**) HLA-DR protein expression was decreased. (**G**) HLA-DQ protein expression of CD14^+^CD86^+^ M1 monocyte was increased with CSE or CSE+HDM stimuli, while (**H**) HLA-DR protein expression of M1 monocyte was decreased with CSE stimulus. * *p* < 0.05 compared with normal culture medium control (NC).

The study investigated the changes in protein expressions of HLA-DQ and HLA-DR in response to environmental stimuli in vitro. Peripheral blood mononuclear cells (PBMCs) from four HS were exposed to different conditions: normal medium control (NC), CSE, HDM, or a combination of CSE and HDM (CSE+HDM). After 24 h of stimulation, both HLA-DQ and HLA-DR protein expression levels were elevated on Th and Tc cells treated with CSE or CSE+HDM compared to NC (*p* < 0.05). However, no significant changes were observed in response to HDM alone (Figure 7A–D). On monocytes, HLA-DQ expression was up-regulated on both M2a and M1 subsets following exposure to CSE or CSE+HDM, while HLA-DR expression was reduced under the same conditions (Figure 7E–H).

#### 3.8.2. Increased HLA-DR Protein Expression with CSE, HDM, and LPS Mixture Stimuli in M2 Polarized THP-1 Cells or M1 Polarized THP-1 Cells with or Without Additional Stimuli

**Figure 8:**
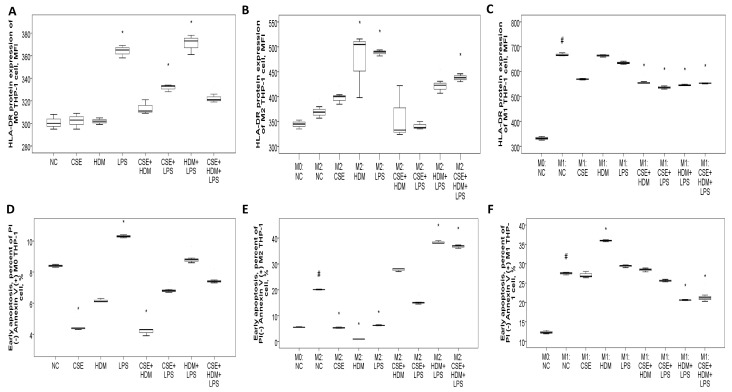
HLA-DR protein expression, early apoptosis, and bactericidal function of THP-1 cells in response to cigarette smoke extract (CSE), house dust mite (HDM), and lipopolysaccharide (LPS) stimuli in vitro. (**A**) HLA-DR protein expression of naïve M0 THP-1 cells was increased with LPS, CSE+LPS, or HDM+LPS stimuli versus normal culture medium control (NC). (**B**) HLA-DR protein expression of M2 THP-1 cells was not changed versus M0 status, but increased with HDM, LPS, or CSE+HDM+LPS stimuli. (**C**) HLA-DR protein expression of M1 THP-1 cells was increased versus M0 status, but decreased with CSE+HDM, CSE+LPS, HDM+LPS, or CSE+HDM+LPS stimuli versus NC. (**D**) Early apoptosis (percent of annexin V (+) PI (−) cells) of M0 THP-1 cells was decreased with CSE or CSE+HDM stimuli, but increased with LPS stimulus. (**E**) Early apoptosis of M2 THP-1 cells was increased compared with M0 status and further increased with HDM+LPS or CSE+HDM+LPS stimuli, but decreased with CSE, HDM, or LPS stimuli. (**F**) Early apoptosis of M1 THP-1 cells was increased compared with M0 status and the HDM stimulus, but decreased with HDM+LPS or CSE+HDM+LPS stimuli. ^#^ *p* < 0.05, compared between M0 and M1/M2. * *p* < 0.05, compared with norma culture medium control (NC).

**Figure 9:**
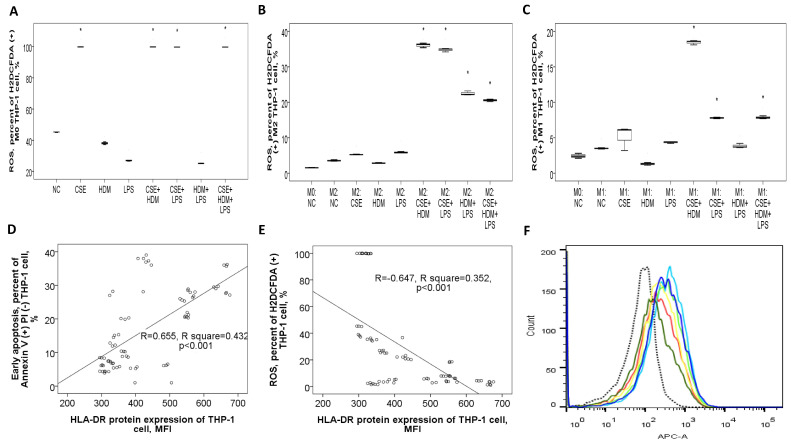
Bactericidal function and its correlation with HLA-DR protein expressions of THP-1 cells in response to cigarette smoke extract (CSE), house dust mite (HDM), and lipopolysaccharide (LPS) stimuli in vitro. (**A**) Reactive oxygen species (ROS) production (percent of H2DCFDA (+) cells) of M0 THP-1 cells was increased with CSE, CSE+HDM, CSE+LPS, or CSE+HDM+LPS stimuli. (**B**) ROS production of M2 THP-1 cells was not significantly changed versus M0 status, but increased with CSE+HDM/CSE+LPS/HDM+LPS/CSE+HDM+LPS stimuli. (**C**) ROS production of M1 THP-1 cells was not significantly changed versus M0 status, but increased with HDM+LPS, CSE+HDM, CSE+LPS, or CSE+HDM+LPS stimuli. HLA-DR protein expression of THP-1 cells was positively correlated with (**D**) early apoptosis, and negatively with (**E**) ROS production at M0, M1, and M2 statuses. (**F**) A representative histogram showing HLA-DR protein expression determined on M0 THP-1 of NC (black dashed line), M1 polarized THP-1 of NC (light blue line), and M1 polarized THP-1 cells in response to various stimuli (other colors). * *p* < 0.05, compared with norma culture medium control (NC).

To investigate the changes in gene/protein expression and biological functions such as apoptosis and bactericidal activity of the HLA-DR molecule, as well as to evaluate the role of M1/M2 polarization in this process, human monocytic THP-1 cells were exposed to CSE, HDM, LPS, or their combinations under M0, M1, or M2 conditions. On naïve M0 THP-1 cells, HLA-DR protein expression was elevated after 24 h of treatment with LPS or a combination of CSE+LPS, HDM+LPS, or CSE+HDM+LPS (Figure 8A). Following M2 polarization induced by IL-4 and IL-13 treatment, HLA-DR protein expression showed no change compared to the M0 state but increased significantly with stimulation from HDM, LPS, or a mixture of CSE+HDM+LPS compared to the negative control (Figure 8B). Meanwhile, after M1 polarization through treatment with IFN-γ and LPS, HLA-DR protein expression was elevated relative to the M0 state but reduced upon stimulation with combinations such as CSE+HDM, CSE+LPS, HDM+LPS, or CSE+HDM+LPS versus NC (Figure 8C and Figure 9F). Overall, HLA-DR protein expression was consistently higher in M1-polarized THP-1 cells compared to M2-polarized THP-1 cells under various conditions.

#### 3.8.3. Apoptosis Proportions and ROS Production of THP-1 Cells Varied with Different Stimuli and at Different Polarization Statuses

Early apoptosis was reduced under CSE or CSE+HDM exposure but increased with LPS stimulation or M1/M2 polarization, as shown in Figure 8D. Following M2 polarization, early apoptosis decreased with CSE/HDM/LPS exposure but increased with HDM+LPS and CSE+HDM+LPS compared to NC, as illustrated in Figure 8E. In contrast, M1 polarization led to increased early apoptosis with HDM exposure and decreased apoptosis with HDM+LPS and CSE+HDM+LPS stimuli, as seen in Figure 8F.

ROS production in M0 THP-1 cells was elevated in response to CSE, CSE+HDM, CSE+LPS, and CSE+HDM+LPS compared to NC, as presented in Figure 9A. After M2 polarization, ROS levels rose in response to CSE+HDM, CSE+LPS, HDM+LPS, and CSE+HDM+LPS exposures versus NC, as presented in Figure 9B. Similarly, under M1 polarization, ROS production increased with CSE+HDM, CSE+LPS, and CSE+HDM+LPS compared to NC, as shown in Figure 9C. Overall, HLA-DR protein expression demonstrated a positive correlation with early apoptosis (Figure 9D) and a negative correlation with ROS production (Figure 9E).

#### 3.8.4. Increased HLA-DRA Gene Expression in M0 THP-1 Cell and Increased HLA-DRB1 Gene Expression in M1 Polarized THP-1 Cell in Response to CSE Stimulus

At the naïve M0 stage, *HLA-DRA* gene expression in THP-1 cells increased following HDM or LPS stimulation but decreased when exposed to CSE+HDM, CSE+LPS, HDM+LPS, or CSE+HDM+LPS combinations. After M2 polarization, *HLA-DRA* gene expression increased in response to CSE, HDM, or LPS stimuli. However, after M1 polarization, *HLA-DRA* expression consistently decreased regardless of the seven stimuli tested (refer to Supplementary Figure S5A,C,E). At either the M0 stage or after M2 polarization, *HLA-DRB1* gene expression in THP-1 cells was elevated with HDM or LPS stimulation. Conversely, after M1 polarization, *HLA-DRB1* gene expression was specifically increased following CSE stimulation (refer to Supplementary Figure S5B,D,F).

Similarly, HLA-DQA1 gene expression increased in response to HDM or LPS stimuli at both M0 and M2 stages. However, it decreased under HDM+LPS, CSE+HDM, CSE+LPS, and CSE+HDM+LPS conditions following M1 polarization (see Supplementary Figure S6A,C,E). As for HLA-DQB1 expression, it rose with LPS stimulation at M0, with HDM or LPS at M2, and with CSE stimulation at M1. Nonetheless, it decreased under CSE+HDM, CSE+LPS, HDM+LPS, or CSE+HDM+LPS conditions irrespective of the polarization state (refer to Supplementary Figure S6B,D,F).

## 4. Discussion

### 4.1. Overview of the Main Findings

After adjusting for age, sex, BMI, and comorbidities, HLA-DR protein expression levels on blood M2a monocytes and helper T cells were found to be higher in both the COPD-only and ACO groups compared to the HS or asthma groups. These levels returned to the normal range after 1 year of medical treatment aligned with the GOLD guidelines. Furthermore, elevated HLA-DR expression was linked to poorer pulmonary function, more frequent exacerbations, and a higher symptom burden. In vitro experiments revealed that HLA-DR expression on M2-polarized THP-1 cells increased when stimulated with a combination of CSE, HDM, and LPS. This increase was associated with heightened apoptosis and reduced bactericidal function.

### 4.2. Increased HLA-DR Expression May Play a Critical Role in Smoking, Allergy, and Microorganism-Related Airway Remodeling in the Circumference of M2 or M1 Polarization

The study reinforces earlier findings of heightened HLA-DR expression in helper T cells, cytotoxic T cells, and B cells in either PBMCs or bronchoalveolar lavage fluid from COPD patients with cachexia or current smokers. Conversely, a decrease in HLA-DR expression was observed with the use of long-acting bronchodilators or anti-inflammatory macrolide treatments [32,33,34]. For example, the use of a LAMA, such as tiotropium, as an add-on therapy to a LABA, such as formoterol, has been shown to reduce the percentage of CD4^+^CD25^+^ regulatory T cells and lower HLA-DR expression in airway lymphocytes of patients with COPD [35].

This study provides, for the first time, clear evidence that an increase in HLA-DR expression is observed on blood M2a monocytes and in patients with ACO, but not in asthma-only patients. This increase is linked with specific clinical phenotypes such as reduced FEV1%predicted value, decreased DLCO% predicted value, shorter walking distance% predicted value, lower maximum inspiratory pressure, higher symptom burden, and more frequent exacerbations. In vitro experiments revealed that HLA-DR expression on Th and Tc cells was up-regulated by CSE or CSE+HDM stimuli, but not by HDM alone. Similarly, the HLA-DR protein expression on THP-1 cells increased following exposure to a combined CSE+HDM+LPS mixture after M2 polarization, or after M1 polarization with or without additional stimuli. Furthermore, HLA-DR protein expression on THP-1 cells showed a positive correlation with apoptosis and a negative correlation with ROS production, potentially contributing to more frequent exacerbations and a decline in lung function. Notably, M2 polarization has been reported to increase in smoking or fine particulate matter-related COPD and appears to play a role in disease progression by promoting airway remodeling and reducing bactericidal activity [36,37]. Conversely, allergic asthma triggered by house dust mites exhibits M2 polarization, while non-allergic asthma induced by farm dust extract is associated with M1 polarization [38]. M2 polarization is associated with scleroderma and tumor progression, whereas M1 polarization is connected to conditions such as atherosclerosis, inflammatory bowel disease, rheumatoid arthritis, and obesity [39]. In accordance with our findings, older adults exhibited a higher proportion of non-classical monocyte subsets, along with elevated levels of HLA-DR, adhesion and migration molecules, and increased production of tumor necrosis factor-α [40]. A significant presence of CD11b^+^CD14^+^CD16^-^HLA-DR-nitric oxide-producing myeloid-derived regulatory cells was observed in the airways of patients with mild asthma, whereas such cells were not detected in patients with COPD or in healthy control subjects [41]. A recent single-cell RNA sequencing analysis found that pediatric patients with HDM-sensitized asthma displayed increased expression of the *HLA-DRA* and *HLA-DRB1* genes in their PBMCs. Additionally, it identified unique gene expression profiles in antigen-presenting cells compared to those observed in non-HDM-sensitized asthma cases [42]. Overall, the findings imply that heightened HLA-DR expression might be a key factor in smoking, allergen, and microorganism-induced airway remodeling, but only when multiple environmental stimuli are present around M2 or following M1 polarization. Additionally, we examined changes in HLA-DR expression under various conditions, observing a consistent down-regulation after a 1-year follow-up. This indicates the absence of subgroup differences. However, since the patients received multiple medications simultaneously, it was not possible to identify which treatment had the greatest impact on this outcome. Furthermore, all patients treated at the clinics were encouraged to reduce or quit smoking as much as possible, though the exact reduction in cigarette consumption during the follow-up period remains unclear. Conversely, recent research consistently shows reduced HLA-DR expression in blood monocytes and dendritic cells among critically ill COVID-19 patients, pointing to a state of immunosuppression [43]. The overlap between the implementation of infection–transmission preventive measures and the decline in COPD exacerbations during the COVID pandemic suggests that individual protective measures may be one of the factors contributing to this simultaneous reduction [44]. Based on our findings, we hypothesize that the decreased HLA-DR expression observed in the majority of patients with COVID infection may partially explain the reduced frequency of exacerbation events in COPD patients.

### 4.3. Both External Stimuli and M1/M2 Polarization Status May Further Affect HLA-DR/-DQ Expressions and Immune Cell Maturation, Which in Turn Lead to Altered Apoptosis and Bactericidal Functions

An important finding of this study is the observed increase in *HLA-DRA* and *HLA-DRB1* gene expressions in PBMCs from COPD patients, as well as in response to HDM or LPS stimuli at the M0 or M2 status of THP-1 cells. Interestingly, after M1 polarization, *HLA-DRA* gene expression decreased regardless of the stimulus, while *HLA-DRB1* gene expression increased specifically with CSE stimulation. Similarly, *HLA-DQA1* and *HLA-DQB1* gene expressions also showed increased levels in response to HDM/LPS stimuli at the M0 or M2 statuses. However, following M1 polarization, *HLA-DQA1* expression declined with all stimuli except CSE, whereas *HLA-DQB1* expression increased with CSE but decreased when exposed to two or three combined stimuli. These fluctuations in HLA-DR/DQ expression suggest an interplay between genetic variants and environmental factors, which could potentially contribute to the pathogenesis of COPD, asthma, and ACO. Additionally, as previously noted, various genetic polymorphisms in *HLA-DQ* and *HLA-DR* genes have been linked to susceptibility or resistance to asthma and COPD [13,24]. Furthermore, cell surface HLA-II levels are governed by the interplay between individual gene transcriptional activity, protein synthesis, heterodimer assembly, translocation from endosomes to plasma membrane, and the rate of HLA-II internalization, recycling, and degradation [45]. Our research suggests that external stimuli, along with the M1/M2 polarization status, can significantly influence HLA-DR/-DQ protein expressions and the maturation of immune cells. These changes may subsequently impact apoptosis, oxidative stress levels, and bactericidal activity.

### 4.4. Compatible with the Results in Previous Studies, ACO Patients Had More Frequent Exacerbations and Higher Symptom Burden than Pure COPD Patients in the Current Study

The current study found that ACO patients experienced more total and moderate exacerbation events, both in the year prior to the study (reported at visit 1) and in the subsequent year (recorded at visit 2), compared with patients with COPD only. Additionally, ACO patients had higher CAT symptom scores than their COPD-only counterparts. These results align with earlier research indicating that ACO patients tend to face more frequent exacerbations, a heavier symptom burden, and reduced quality of life [46]. Absolute eosinophil counts were found to be higher in both ACO and asthma-only patients compared to COPD-only patients. Additionally, the percentage of inflammatory eosinophils (CD125^+^Siglec8^+^) returned to normal levels following one year of medical treatment in COPD and ACO patients. These findings align with a recent systematic review indicating that ACO patients exhibit higher blood eosinophil levels than those with pure COPD [47]. Additionally, it has been reported that patients with asthma had a higher proportion of inflammatory eosinophils in the blood as compared with COPD patients or smokers without COPD [48].

### 4.5. Limitations and Strengths

The current study has several limitations that should be acknowledged. Firstly, clinical variables such as age, gender, BMI, and comorbidities may influence HLA-DR/DQ expression levels. To address this, we performed multivariate linear regression analyses to minimize the impact of these confounding factors and calculate adjusted *p*-values. Secondly, factors like polymorphisms or epigenetic modifications of individual genes might also affect HLA-DR/DQ expression, which were not assessed in this study. Nonetheless, we employed an in vitro cell culture model and demonstrated that HLA-DR expression on THP-1 cells was elevated in response to a combination of CSE, HDM, and LPS under M2 conditions, or after M1 polarization with or without additional stimuli. These findings suggest that environmental triggers and M1/M2 polarization states predominantly regulate HLA-DR expression, subsequently influencing apoptosis and bactericidal functions.

Thirdly, it was observed that ACO patients experienced more frequent moderate exacerbations and exhibited a higher symptom load compared to pure COPD patients. However, no significant differences in HLA-DR expression were observed between these two groups. This disparity indicates that mechanisms unrelated to MHC class II signaling might be driving the pathogenesis of eosinophilic COPD or ACO. On the other hand, blood M2a monocytes in ACO and pure COPD patients showed higher HLA-DR expression levels than in asthma patients or HS; these levels normalized after 1 year of medical treatment. This suggests that HLA-DR could serve as a biomarker for distinguishing smoke-related COPD or ACO from pure asthma and for monitoring treatment response in the former two conditions.

Fourthly, the study was based on data from a single-center cohort, lacked power and sample size analyses, and did not include replication or validation in external datasets. Further research involving external validation cohorts is necessary to confirm these findings before they can be clinically applied. Fifthly, the CSE, HDM, and LPS concentrations used in the in vitro experiments were based on previous studies and may not fully reflect physiologically relevant levels. However, we determined optimal concentrations using WST-1 assays to ensure that cell viability was not compromised by more than 30–40%. Sixthly, dendritic cells are predominantly found in the skin, intestines, and lymph nodes, with only a small number of immature ones present in the bloodstream. Due to challenges in obtaining dendritic cells in clinical settings, their use as a monitoring tool is limited. In contrast, blood immune cells are more readily accessible and easier to isolate, making them valuable for studying their role in the pathogenesis of chronic airway diseases. Finally, while this study proposes a mechanistic role for HLA-DR in the progression of ACO and COPD, this is not decisively supported by the functional data from THP-1 cells. However, we observed that HLA-DR expression displayed a positive correlation with apoptosis and a negative correlation with ROS production. Additionally, apoptosis increased significantly following M1/M2 polarization, whereas ROS production decreased. This implies that changes in HLA-DR expression in response to environmental stimuli could be influenced by M1/M2 status and, in turn, impact cell apoptosis. Additional studies investigating the effects of MHC class II signaling in bronchial epithelial cells or airway smooth muscle cells are essential to better understand the underlying disease mechanisms.

## 5. Conclusions

In summary, increased HLA-DR expression on blood M2a monocytes and helper T cells is observed at diagnosis in both COPD and ACO patients compared to healthy non-smokers with minimal or no allergic attributes. These elevated levels return to normal after one year of medical treatment. The initial up-regulation of HLA-DR expression correlates with clinical phenotypes of chronic inflammatory disease, including reduced pulmonary function, higher symptom burdens, and increased frequency of exacerbations. Elevated HLA-DR expression on M2a monocytes of COPD and ACO patients is linked to more severe airflow limitations. Moreover, HLA-DR expression is up-regulated in response to LPS, HDM, or a mixture of CSE, HDM, and LPS in THP-1 cells after M2 polarization, or after M1 polarization, accompanied by increased apoptosis and impaired bactericidal function. This suggests a pivotal role for HLA-DR in cigarette smoke, allergen, and microorganism-induced airway remodeling. Additionally, monitoring HLA-DR expression on blood M2a monocytes, neutrophils, and T cells could serve as a predictive marker for early identification of COPD and ACO patients at high risk of frequent exacerbations and lung function deterioration. It may also function as a tool for evaluating treatment responses. Further research using an external validation cohort is necessary to confirm these clinical applications. Additionally, studies focusing on the impact of MHC class II signaling in bronchial epithelial cells or airway smooth muscle cells are needed to elucidate the underlying mechanisms.

## Figures and Tables

**Table 1:** Demographic and baseline characteristics of the 116 study participants.

	Healthy Subjects N = 18	COPD Only N = 41	Asthma and COPD Overlap N = 37	Asthma Only N = 20	*p* Value
Age, years	65 ± 11.4	70.7 ± 11.2	68.8 ± 9.8	60 ± 14.4	0.006
Male Gender, n (%)	12 (66.7)	35 (85.4)	36 (97.3)	8 (40)	<0.001
Cigarette smoke, pack-years	5.1 ± 8.4	38.4 ± 25.1	40.7 ± 24.5	1.8 ± 5.6	<0.001
Smoke history, n (%)					<0.001
Current heavy smoker	0 (0)	21 (51.2)	25 (67.6)	0 (0)	
Remote heavy smoker	6 (33.3)	20 (48.8)	12 (32.4)	0 (0)	
Charlson Comorbidity Index	3.9 ± 1.2	5.6 ± 2.5	5.2 ± 1.8	4.0 ± 2.1	0.005
COPD assessment test (CAT)	7.5 ± 6.7	13.1 ± 7.2	17.1 ± 7.4	16.3 ± 9.9	<0.001
mMRC, point	0.6 ± 0.9	1.3 ± 0.8	1.3 ± 0.9	1.1 ± 0.7	0.036
Absolute eosinophil count, cells/ml	113.4 ± 57.5	213.2 ± 118	295.5 ± 214.9	311.1 ± 232.1	0.001
Post-BD FEV1/FVC, % predicted	83.1 ± 5.1	67.8 ± 12.5	66.3 ± 11.5	78.7 ± 11.2	<0.001
Post-BD FEV1, % predicted	104.8 ± 11.1	75.2 ± 23.5	73.5 ± 21.3	90.4 ± 22.4	<0.001
Post-BD FEF_25–75%_, % predicted	94.2 ± 25.3	44.5 ± 24.1	42.8 ± 21.5	71.2 ± 37.4	<0.001
DLCO, % predicted	82.4 ± 18.2	63.6 ± 23.1	66.5 ± 19.7	74 ± 20.3	0.032
Alveolar Volume (VA), % predicted	82.4 ± 10.9	64 ± 22.1	73.2 ± 14	80.3 ± 17.6	0.003
Exacerbation events/year, n	0.5 ± 0.8	2.3 ± 1.5	3.8 ± 1.8	2.6 ± 1.9	<0.001
Mild exacerbation, n	0.5 ± 0.8	1.9 ± 1.1	2.4 ± 1.1	1.8 ± 1.2	<0.001
Moderate, n	0 ± 0	0.3 ± 0.6	1.2 ± 1.1	0.8 ± 1	<0.001
Severe, n	0 ± 0	0 ± 0.2	0.2 ± 0.5	0 ± 0	0.044

COPD = chronic obstructive pulmonary disease; mMRC = modified Medical Research Council; DLCO = diffusion capacity of carbon monoxide; FEV1 = forced expiratory volume in 1 s; FVC = forced vital capacity; FEF_25–75%_ = forced mid-expiratory flow.

**Table 2:** Regression coefficients and confidence intervals in multivariable linear regression models for HLA-DR protein expressions on various blood immune cells.

	HLA-DR on Th Cell, MFI	HLA-DR on Tc Cell, MFI	HLA-DR on M2a Monocyte, MFI	HLA-DR on M1 Monocyte, MFI	HLA-DR on B Cell, MFI	HLA-DR on Neutrophil, MFI
Age, years	12.6 (−2.76 to 27.96)	4.8 (−4.04 to 13.64)	61.37 * (11.56 to 111.18)	113.73 (−0.29 to 227.76)	−39.82 (−186.7 to 107.05)	0.54 (−21.89 to 22.98)
Sex	328.15 (−75.66 to 731.98)	−8.58 (−240.99 to 223.81)	1178.54 (−300 to 2657.1)	−157.23 (−3153.5 to 2839.0)	1233.59 (−2625.9 to 5093.1)	363.28 (−226.38 to 952.84)
Smoking exposure, pack-years	3.0 (−3.41 to 9.42)	2.04 (−1.65 to 5.73)	29.3 * (7.57 to 51.03)	22.7 (−24.91 to 70.32)	68.29 * (6.95 to 129.63)	11.18 * (2.78 to 19.58)
Charlson comorbidity index	27.33 (−54.36 to 109.02)	23.24 (−23.77 to 70.26)	−73.61 (−372.7 to 225.5)	−104.79 (−710.9 to 501.3)	−111.88 (−892.68 to 668.91)	3.48 (−115.81 to 122.77)

Th = helper T, Tc = cytotoxic T, MFI = mean fluorescence intensity. * *p* < 0.05.

**Table 3:** Specific medicines for the 30 patients with chronic obstructive pulmonary disease (COPD) or asthma and COPD overlap (ACO) between visit 1 (at the first study enrollment) and 2 (after 1-year medical treatment).

Medicine	COPD Patients, n = 14	ACO Patients, n = 16	*p* Value
Dual bronchodilator	9 (64.3%)	4 (25%)	0.03
Triple therapy	5 (35.7%)	12 (75%)	0.03
Oral corticosteroid	2 (14.3%)	3 (18.8%)	0.743
theophylline	7 (50%)	10 (62.5%)	0.491
macrolide	2 (14.3%)	3 (18.8%)	0.743

## Data Availability

The original contributions presented in this study are included in the article and Supplementary Materials. Further inquiries can be directed to the corresponding authors.

## Supplementary Materials

The following supporting information can be downloaded at https://www.mdpi.com/article/10.3390/antiox14121507/s1. Supplementary Figure S1: Clinical characteristics of patients with pure COPD, pure asthma, and asthma and COPD overlap (ACO); Supplementary Figure S2: Diagrams showing representative flowcytometry plots and histograms of blood immune cells and THP-1 cells; Supplementary Figure S3: Correlations between HLA-DR protein expressions of blood immune cells and age/smoke exposure amount; Supplementary Figure S4: HLA-DRA/DRB1/DQA1 gene expressions and their correlations with clinical characteristics; Supplementary Figure S5: HLA-DRA/DRB1 genes expressions of THP-1 cells; Supplementary Figure S6: HLA-DQA1/DQB1 gene expressions of THP-1 cells; Supplementary Table S1: Primer sequences used in the quantitative RT-PCR assays. Supplementary Table S2: Additional demographic and baseline characteristics of the 116 study participants.

## Abbreviations

The following abbreviations are used in this manuscript:

COPD	chronic obstructive pulmonary disease
ACO	asthma and COPD overlap
HLA	human leukocyte antigen
MHC	major histocompatibility complex
FEV1	forced expiratory volume in 1 s
FVC	forced vital capacity
BD	bronchodilator
6MWT	6-min walking test
RT-PCR	reverse transcriptase polymerase chain
IgE	immunoglobulin E
CSE	cigarette smoke extract
HDM	house dust mite
LPS	lipopolysaccharide
CAT	COPD assessment test
mMRC	Modified Medical Research Council
DLCO	diffusion capacity of carbon monoxide
MFI	mean fluorescence intensity
PI	propidium iodide
ROS	reactive oxygen species
VA	alveolar volume
