# Supplementary material for: Increased HLA-DR Expression on M2a Monocytes and Helper T Cells in Patients with COPD and Asthma–COPD Overlap Contributes to Disease Severity via Apoptosis and ROS
Source: Antioxidants (Basel). 2025 Dec 16;14(12):1507. doi: 10.3390/antiox14121507 (PMC12729834; doi:10.3390/antiox14121507)
Supplement: Supplementary file 1 [file antioxidants-14-01507-s001.zip › antioxidants-3994671-supplementary.pdf]

Increased HLA-DR Expression on M2a Monocytes and Helper T Cells in Patients with COPD and Asthma–COPD Overlap Contributes to Disease Severity via Apoptosis and ROS

Yung-Che Chen [1,2,3], Kuo-Tung Huang [1,3], Chiu-Ping Lee [1], Po-Yuan Hsu [1,4], Yu-Ping Chang [1], Chao-Chien Wu [1], Sum-Yee Leung [1,3], Chang-Chun Hsiao [2,*] and Meng-Chih Lin [1,4,*]

Supplementary Material

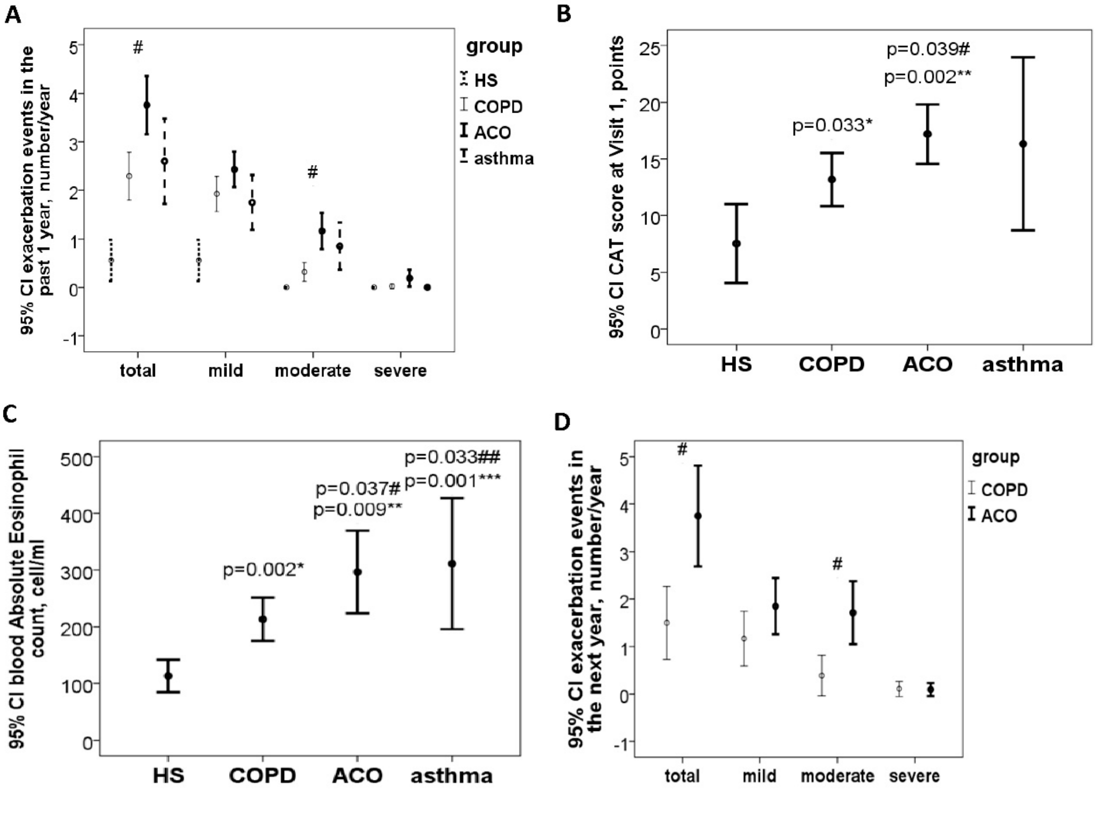

Supplementary Figure S1. Clinical characteristics of patients with pure COPD, pure asthma, and asthma and COPD overlap (ACO). (A) Total and moderate exacerbation events were increased in ACO patients versus pure COPD patients in the past one year (visit 1). (B) COPD assessment test (CAT) scores were higher in ACO

patients versus pure COPD patients. (C) Blood absolute eosinophil counts were higher in ACO and asthma patients versus either pure COPD or healthy subject (HS) group. (D) Total and moderate exacerbation events were increased in ACO patients versus pure COPD patients in the next one year (visit 2).

*compared between COPD and HS

**compared between ACO and HS

***compared between asthma and HS

#compared between ACO and COPD

##compared between asthma and COPD

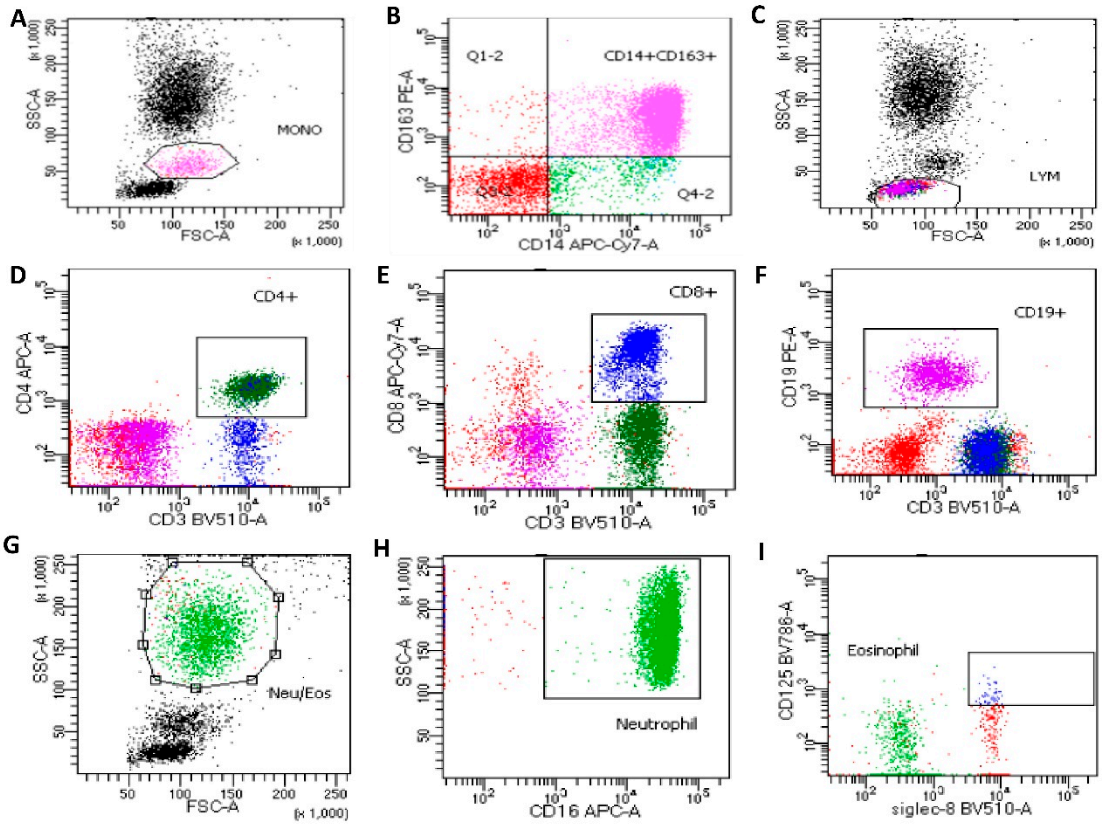

Supplementary Figure S2. **Diagrams showing representative flowcytometry plots and histograms of blood immune cells and THP-1 cells.** (A) 2-D scatter plot reveals three classifications of blood immune cells based on morphology by plotting forward scatter against side scatter data. (B) Blood M2A monocyte was further gated by the cell surface expressions of both CD14 and CD163. (C) Blood helper T cell was gated by the cell surface expressions of both CD3 and CD4. (D) Blood cytotoxic T cell was gated by the cell surface expressions of both CD3 and CD8. (E) Blood B cell was identified based on the cell surface expression of CD19. (F) Blood neutrophil was identified based on the cell surface expression of CD16. (G) HLA-DR protein expression was determined on blood $CD3^{+}CD8^{+}$ cytotoxic T cell populations. (H) HLA-DR protein expression was determined on $CD19^{+}$ B cell populations. (I) HLA-DR protein expression was determined on $CD16^{+}$ neutrophil populations.

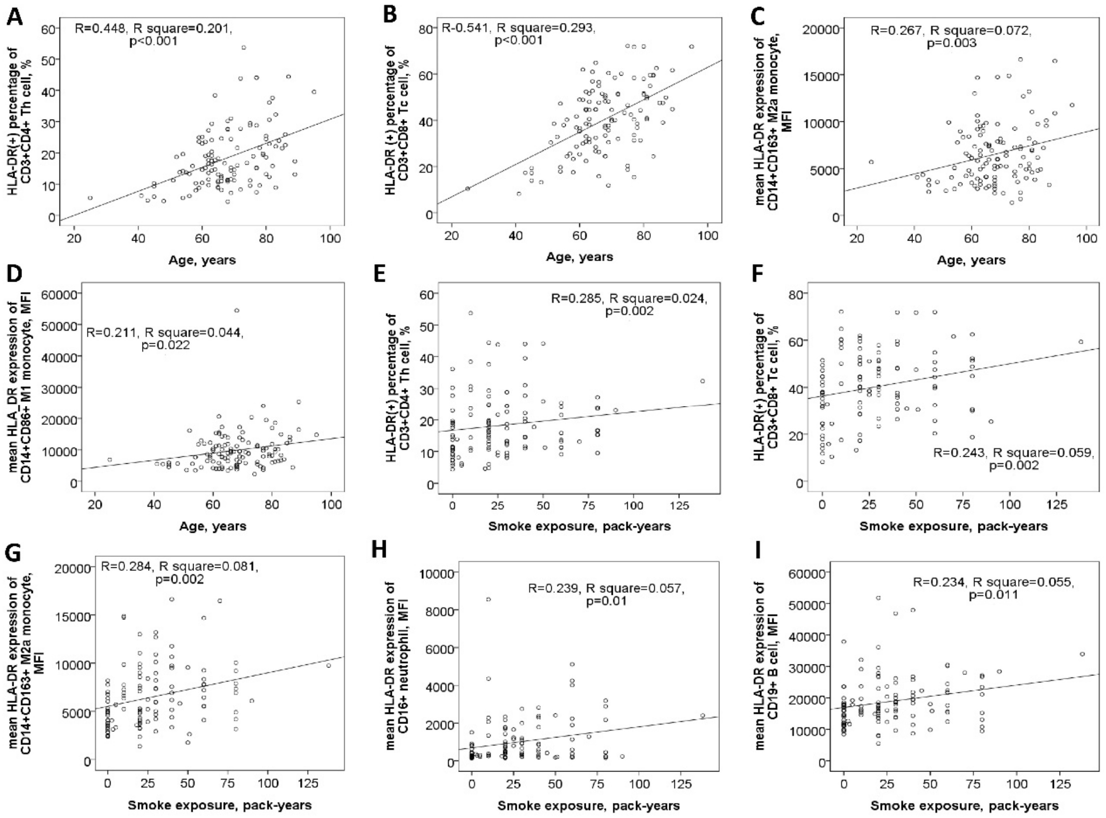

Supplementary Figure S3. Correlations between HLA-DR protein expressions of blood immune cells and age/smoke exposure amount. Age was positively correlated with HLA-DR protein expressions of blood (A) $CD3^+CD4^+$helper T cell, (B) $CD3^+CD8^+$cytotoxic T cell, (C) $CD14^+CD163^+$M2a monocyte, and (D) $CD14^+CD86^+$M1 monocyte. Cigarette smoke exposure pack-years were positively correlated with HLA-DR protein expressions of blood (E) helper T cell, (F) cytotoxic T cell, (G) M2a monocyte, (H) $CD16^+$neutrophil, and (I) $CD19^+$B cell.

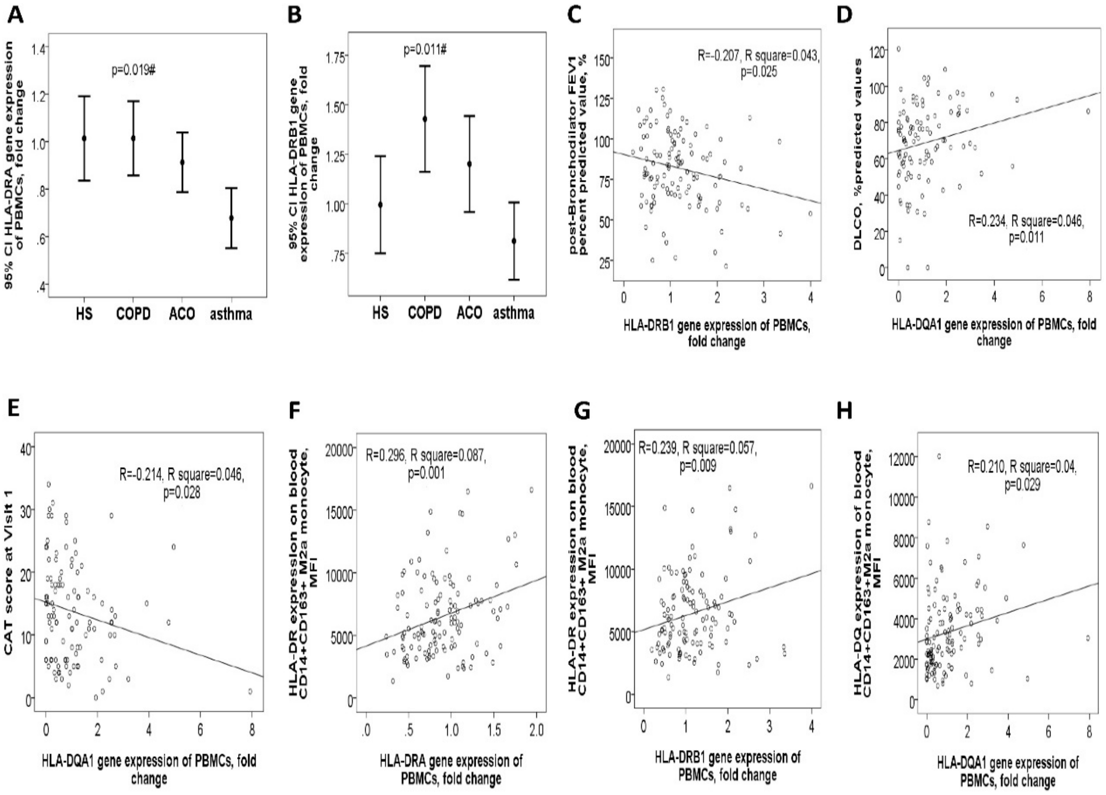

Supplementary Figure S4. ***HLA-DRA/DRB1/DQA1*** **gene expressions and their correlations with clinical characteristics. (A)** *HLA-DRA* and (B) *HLA-DRB1* gene expressions of peripheral blood mononuclear cells were both increased in COPD only patients versus asthma only patients, and (C) the former was negatively correlated with post-BD FEV1 % predicted value. (D) *HLA-DQA1* gene expression was positively correlated with DLCO % predicted value, and (E) negatively correlated with COPD assessment test symptom scores. (F) *HLA-DRA* gene expression was positively correlated with HLA-DR protein expression of blood M2a monocyte. (G) *HLA-DRB1* gene expression was positively correlated with HLA-DR protein expression of blood M2a monocyte. (H) *HLA-DQA1* gene expression was positively correlated with HLA-DQ protein expression of blood M2a monocyte.

#compared between COPD only and asthma only groups

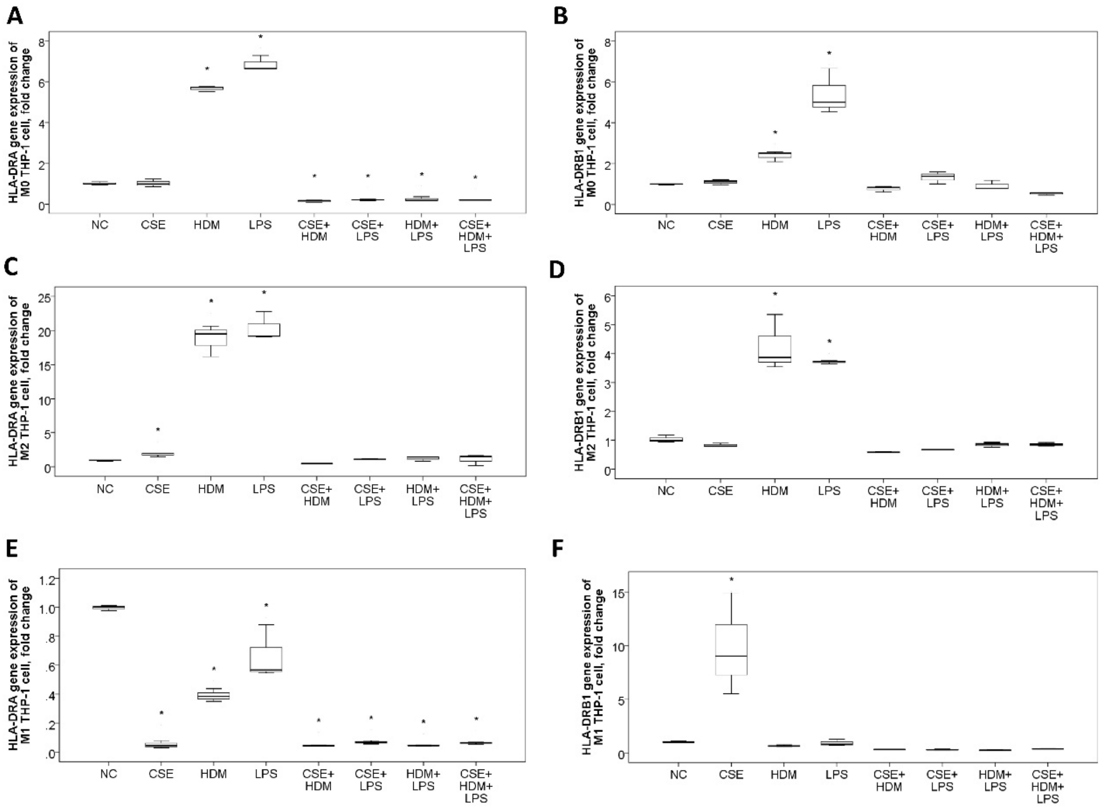

Supplementary Figure S5. ***HLA-DRA/DRB1*** **genes expressions of THP-1 cells.**

HLA-DRA gene expression of THP-1 cell was increased with HDM or LPS stimulus versus NC at (A) M0 or (C) M2 status, but (E) decreased with any stimulus at M1 status. *HLA-DRB1* gene expression of THP-1 cell was increased with HDM or LPS stimulus at (B) M0 or (D) M2 status, while (F) increased only with CSE stimulus at M1 status.

*p<0.05, compared with normal culture medium control (NC)

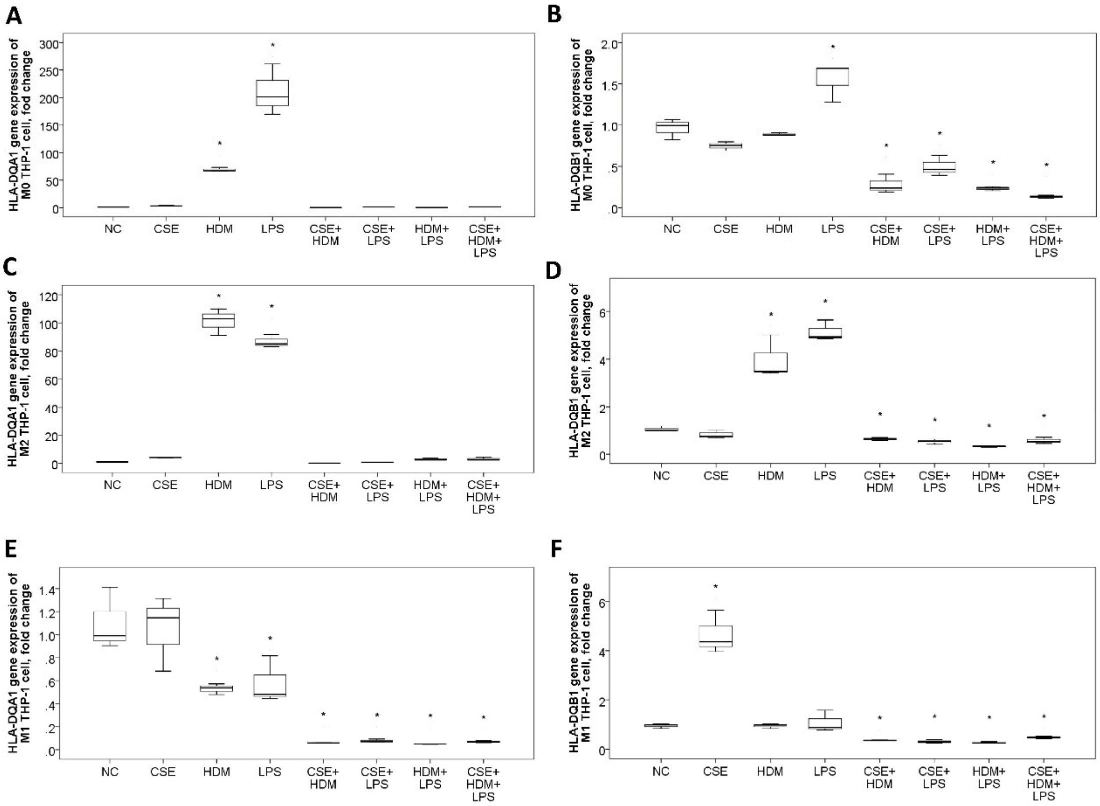

Supplementary Figure S6. *HLA-DQA1/DQB1* gene expressions of THP-1 cells.

HLA-DQA1 Gene expression of THP-1 cell was increased with HDM or LPS stimulus at (A) M0 or (C) M2 status, but (E) decreased with any stimulus except for CSE at M1 status. *HLA-DQB1* gene expression of THP-1 cell was (B) increased with LPS stimulus at M0 and (D) increased with HDM or LPS stimulus at M2 status, but decreased with CSE+HDM, CSE+LPS, HDM+LPS, or CSE+HDM+LPS stimuli at M0 or M2 status. (F) *HLA-DQB1* gene expression increased with CSE stimulus but decreased with CSE+HDM, CSE+LPS, HDM+LPS, or CSE+HDM+LPS stimuli at M1 status.

*p<0.05, compared with normal culture medium control (NC)

Supplementary Table S1. Primer sequences used in the quantitative RT-PCR assays

Gene	Primer Sequence
HLA-DRA	Forward: TCT GGC GGC TTG AAG AAT TTG
	Reverse: GGT GAT CGG AGT ATA GTT GGA GC
HLA-DRB1	Forward: CGG GGT TGG TGA GAG CTT C
	Reverse: AAC CAC CTG ACT TCA ATG CTG
HLA-DQB1	Forward: GCG GGA TCT TGC AGA GGA G
	Reverse: ACT TTG ATC TGG CCT GGA TAG AA
HLA-DQA1	Forward: AGATGAGCAGTTCTACGTGGA
	Reverse: ACGGGAGACTTGGAAAACACT

Supplementary Table S2. Additional demographic and baseline characteristics of the 116 study participants

	Healthy subjects N=18	COPD only N=41	Asthma and COPD overlap N=37	Asthma only N=20	p value
Body mass index, kg/m2	25.2±3.3	25.6±4.5	26.0±3.3	25.7±4.4	0.932
Hypertension, n (%)	10 (55.6)	23 (56.1)	20 (54.1)	8 (40)	0.666
Diabetes mellitus, n (%)	5 (27.8)	12 (29.3)	15 (40.5)	2 (10)	0.118
Allergic rhinitis, n (%)	8 (44.4)	30 (73.2)	26 (70.3)	17 (85)	0.048
Allergic dermatitis, n (%)	1 (5.6)	6 (14.6)	3 (8.1)	1 (5)	0.544
Total immunoglobulin E, IU/ml	63.4±77.6	157.8±208.3	253.4±411.2	168±239.9	0.142
Eosinophil cationic protein, μg/L	6.6±4.1	11.2±11.2	11.7±11.7	62.1±193.1	0.08
6MWT distance, % predicted	94.6±15.9	83.6±19.7	81.5±18.7	85.7±14.6	0.143
MIP, mmHg	102±49.0	76.9±66.4	55.6±102.4	62.6±86.6	0.31
MEP, mmHg	101.6±32.7	114.8±34.6	125.6±42.4	98.6±42.5	0.084
Absolute neutrophil count, cells/ml	4186±2396	5385±6220	4452±1664	5215±3736	0.592

6MWT= 6-minute walking test; MIP= maximum inspiratory pressure (absolute value); MEP= maximum expiratory pressure
